# Prognostic significance of intra-tumoral budding in high-grade serous ovarian carcinomas

**DOI:** 10.1038/s41598-022-07269-2

**Published:** 2022-02-24

**Authors:** Toru Hachisuga, Midori Murakami, Hiroshi Harada, Taeko Ueda, Tomoko Kurita, Seiji Kagami, Kiyoshi Yoshino, Ryosuke Tajiri, Masanori Hisaoka

**Affiliations:** 1 Department of Obstetrics and Gynecology, School of Medicine, University of Occupational and Environmental Health, 1-1, Iseigaoka, Yahatanishi-ku, Kitakyushu, 807-8555 Japan; 2 Department of Pathology and Oncology, School of Medicine, University of Occupational and Environmental Health, 1-1, Iseigaoka, Yahatanishi-ku, Kitakyushu, 807-8555 Japan

**Keywords:** Cancer, Medical research, Oncology

## Abstract

Intra-tumoral budding (ITB) has been well demonstrated to be an independent risk factor for adverse outcomes in colorectal carcinoma. This study investigated the prognostic significance of ITB in high-grade serous ovarian carcinomas (HGSOCs). The medical records and slides of 84 SOCs, including 13 with neoadjuvant chemotherapy (NAC), were retrospectively reviewed. The histopathologic examination with scoring of p53 expression showed them to be 80 HGSOCs and 4 low-grade serous ovarian carcinomas (LGSOCs). ITB was found in 64 (80.0%) of the 80 HGSOCs and 1 (25.0%) of 4 LGSOCs. The presence of ITB in HGSOC was significantly correlated with a higher level of CA125, an advanced 2014 FIGO stage, the presence of Lymph node metastasis, and the presence of lymphovascular space invasion (LVSI). The median progression-free survival (PFS) was 18 months in patients with HGSOC with ITB and 36 months in patients with HGSOC without ITB (P = 0.006), and their median overall survival (OS) was 50 months and 60 months (P = 0.060). The multivariate analysis revealed that ITB was not an independent prognostic factor. ITB is a cost-effective prognostic indicator for patients with HGSOC and ITB in ovarian tumor tissue is considered a useful histological biomarker of the progression of HGSOCs.

## Introduction

Many authors have examined the prognostic significance of histopathologic features for invasive ovarian carcinomas. Although Shimizu and Silverberg^1^ proposed a three-tier grading system, which was based on the dominant architectural pattern, degree of nuclear pleomorphism, and mitotic index, for all ovarian epithelial malignancies, the international collaboration on cancer reporting (ICCR)^2^ recommends that different grading systems should be used for different morphological subtypes.

Ovarian high- and low-grade serous carcinoma, was defined by Malpica et al.^3^ in 2004 and subsequently adopted in the 2014 WHO Classification of Tumours of Female Reproductive Organs^4^. Using this two-tier grading system for serous ovarian carcinomas (SOCs)^3^, cases assigned to the low-grade category were characterized by the presence of mild to moderate nuclear atypia. As a secondary feature, they tended to show up to 12 mitoses/10 high-power fields (HPFs), whereas those in the high-grade category had marked nuclear atypia and as a secondary feature more than 12 mitoses/10 HPFs. Recent molecular genetic studies have shown that LGSOCs demonstrate mutations in KRAS or BRAF and few chromosomal abnormalities. In contrast, HGSOCs harbor mutations in TP53 and are, chromosomally, highly unstable^5^. Immunohistochemical algorithms and prediction models have been proposed for the five major histologic types of epithelial ovarian carcinomas^6^. Optimized p53 immunohistochemistry was reported to be clinically useful as it can exclude the possibility of a LGSOC^7^.

Tumor budding (TB) and its association with disease progression in patients with various solid cancers was first described by Imai in the 1950s^8^. TB is a morphological phenotype representing a destructive stromal invasion and was included as an additional prognostic factor for colorectal carcinoma in the 2019 WHO Classification of Digestive System Tumours^9^. The term intra-tumoral budding (ITB), which is TB found within the main tumor body, was introduced to distinguish this form of budding from the classic peritumoral budding^10^. The reported interobserver variability for assessing TB has ranged from moderate to very good, depending on the study^11^.

The prognostic significance of destructive invasive implants in extra-ovarian tissues has been well studied in serous borderline tumors (SBTs) of the ovary^12^. Such destructive invasion was suggested to be associated with a poor prognosis in patients with stage I endometrioid and mucinous ovarian carcinomas^13^. TB is an independent, unfavorable, prognostic factor for patients with early-stage cervical cancer following radical surgery^14^. However, ITB has not been well described in SOCs.

The present study evaluated the prognostic significance of ITB in HGSOCs, describing the correlation between ITB and other conventional histologic parameters.

## Results

### Distinguishing LGSOC from HGSOC

Among 84 SOC cases, p53 expression was scored as overexpression in 52 cases, complete absence in 25 cases and wild type in 7 cases. A cytoplasmic pattern of p53 expression was not observed in this study. Among seven tumors with wild type of p53 expression, three tumors showing grade 3 nuclear atypia were morphologically indistinguishable from HGSOC with abnormal expression of p53. These tumors were included in HGSOCs. As a result, the 84 SOCs were divided into 80 HGSOCs and 4 LGSOCs.

### Clinicopathologic features

**Figure 1:**
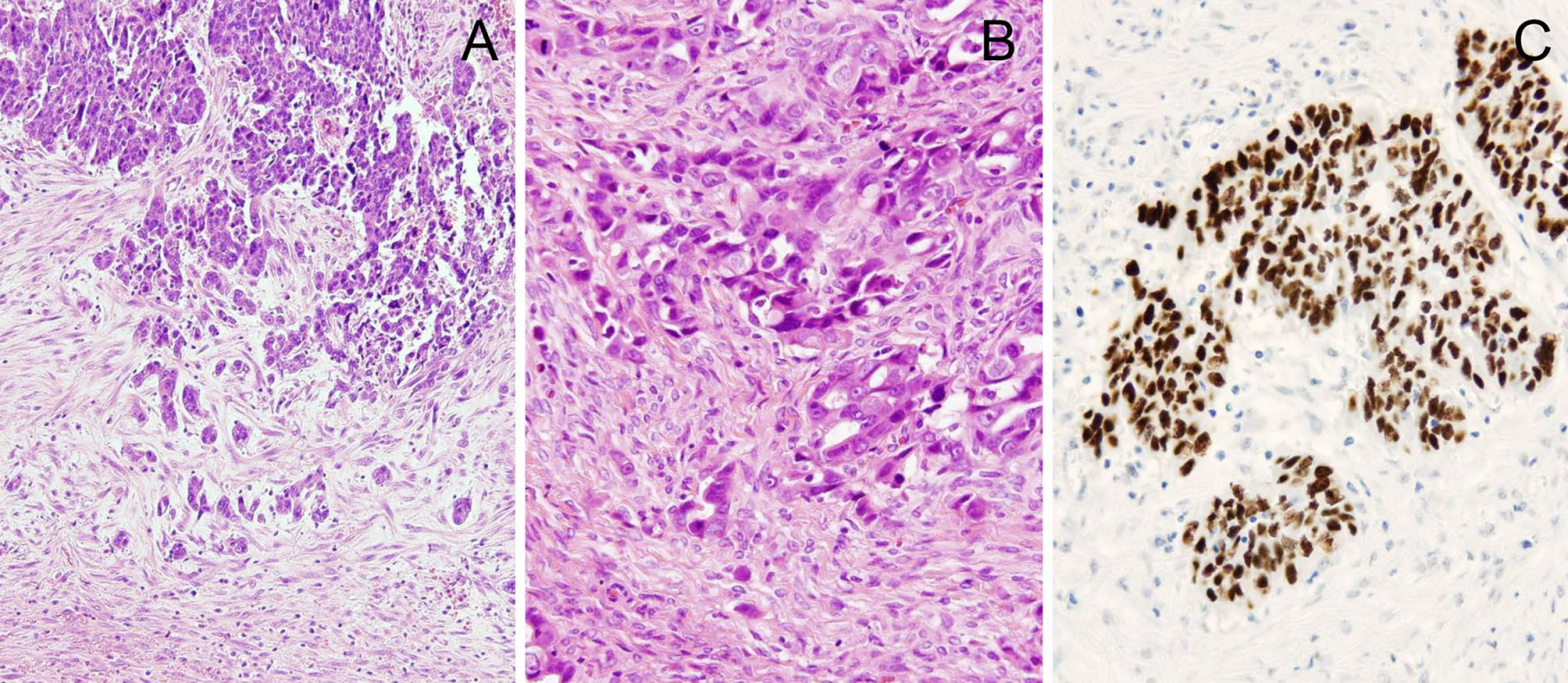
High grade serous ovarian carcinomas with intra-tumoral budding in a 48-year-old patient (**A**). Intra-tumoral budding was defined as a single tumor cell or a cell cluster of up to four tumor cells at the invasive tumor front. Single tumor cells and clusters of small numbers of tumor cells were noted at the invasive edge (**B**). p53 stained section showed aberrant overexpression (**C**).

**Figure 2:**
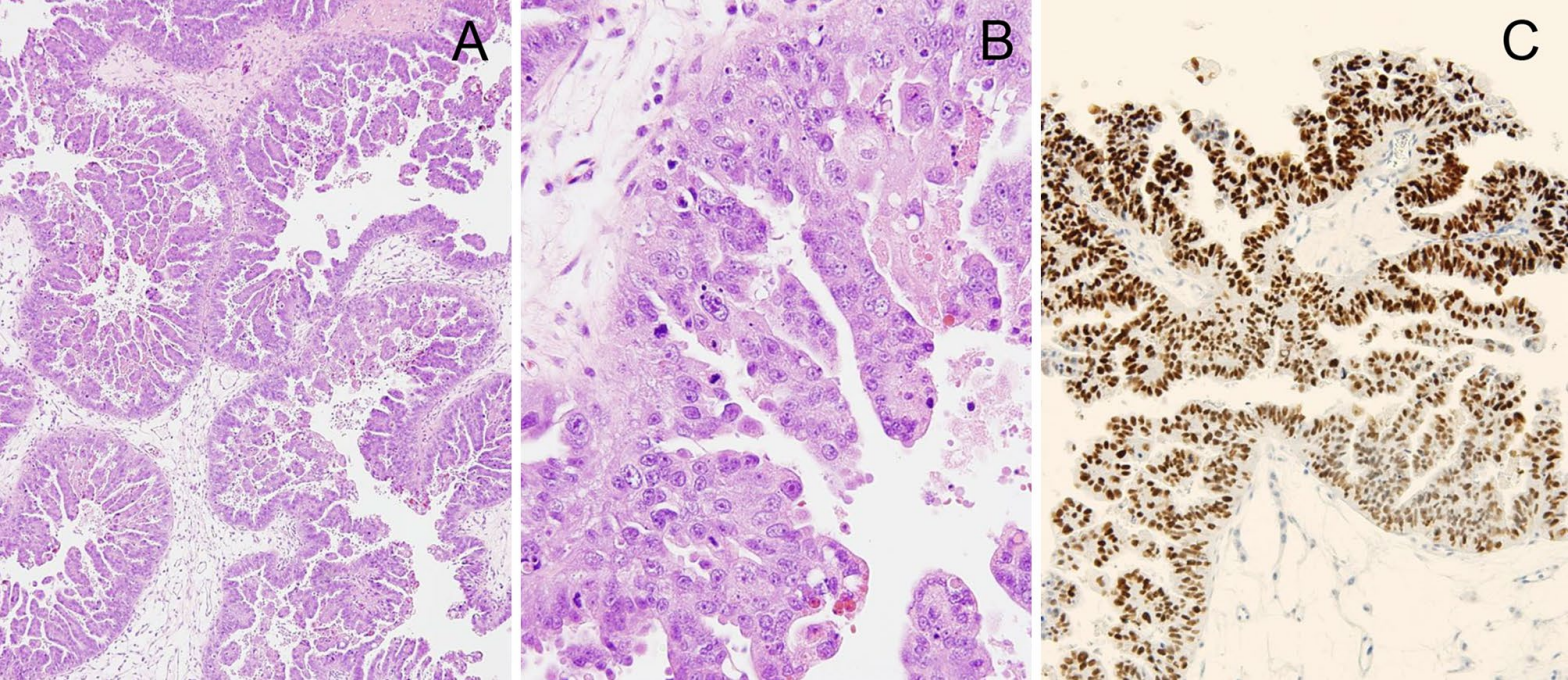
High grade serous ovarian carcinoma in a 49-year-old patient. The stage IC1 tumor showed no intra-tumoral budding and architectural grade 1 (**A**), and nuclear grade 3 and a high number of mitoses (**B**). p53 stained section showed aberrant overexpression (**C**).

The clinicopathologic data of 80 HGSOCs and 4 LGSOCs are shown in Table 1. ITB was found in 64 (80.0%) of the 80 HGSOCs and 1 (25.0%) of 4 LGSOCs. The values of CA125 level (P = 0.013), 2014 FIGO stage (P = 0.002), presence of lymph node metastasis (P = 0.049) and presence of LVSI (P < 0.001) in patients with HGSOC with ITB (Fig. 1) were significantly higher than those in patients with HGSOC without ITB (Fig. 2). The levels of CA125 before initial treatment and after 3cycles of cisplatin-based chemotherapy were measured in 80 and 71 patients with HGSOC and 4 and 4 patients with LGSOC, respectively. The CA125 level after 3 cycles of chemotherapy was shown to be normal in 4 patients with LGSOCs (100.0%), and 13 patients with HGSOC without ITB (100.0%), and 43 (74.1%) of 58 patients with HGSOC with ITB. All 13 patients with HGSOC who had undergone NAC showed ITB. Nine of these 13 HGSOCs showed up to 12 mitoses/10HPFs (HGSOCs with NAC vs. without NAC, P < 0.001). Seven showed the overexpression of p53 and six showed the complete absence of p53 expression. Poly (ADP-ribose) polymerase (PARP) inhibitor and bevacizumab were administered to three and three patients with HGSOC without ITB, and five and eight patients with HGSOC with ITB, respectively.

**Table 1:** Clinicopathologic characteristics of 84 serous ovarian carcinomas.

Variables	HGSOC	LGSOC
Number of cases	80	4
Age (years) median (range)	63 (34–88)	59 (48–66)
Retroperitoneal lymph node sampling	55	4
**Platinum-based chemotherapy**		
Neoadjuvant	13	0
Adjuvant	60	4
**CA125 before initial treatment**		
Median	691.0	206.4
Range	6.0–17,815.2	132.5–8367.0
**2014 FIGO stage**		
I and II	15	2
III and IV	65	2
**Architectural grade**		
1 and 2	52	3
3	28	1
**Nuclear grade**		
1	0	4
2	7	0
3	73	0
Mitoses* median (range)	22 (0–56)	8 (6–12)
**Lymphovascular space invasion**		
Present	47	0
Absent	33	4
**Lymph node metastasis**		
Present	22	2
Absent	33	2
**Intra-tumoral budding**		
Present	64	1
Absent	16	3
**Expression of p53**		
Over expression	52	0
Complete absence	25	0
Wild type	3	4

*HGSOC* high-grade serous ovarian caricinoma, L*GSOC* low-grade serous ovarian carcinoma.

*X 10 high power fields.

### The prognosis

**Figure 3:**
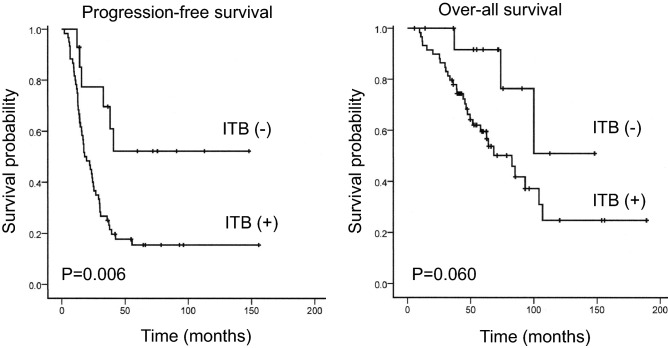
Kaplan–Meier survival analyses of the progression-free and over-all survivals in patients with high-grade serous ovarian carcinoma with and without intra-tumoral budding (ITB).

Figure 3 shows the differential Kaplan–Meier PFS and OS curves of patients with HGSOC for the presence or absence of ITB. The median PFS was 18 months in 60 patients with HGSOC with ITB (range 2 to 156 months) and 36 months in 14 women with HGSOC without ITB (range 12 to 148 months), and their median OS was 50 months (range 6 to 189 months) and 60 months (range 14 to 148 months). The presence of ITB had significantly adverse effect on the PFS of patients with HGSOC (P = 0.006), but had not significantly adverse effect on the OS of patients with HGSOC (P = 0.060).

Table 2 showed univariate and multivariate analyses for the PFS and OS of patients with HGSOC. A univariate analysis showed that the 2014 FIGO stage, the CA125 level before initial treatment, LVSI and ITB were significantly associated with the PFS and that the 2014 FIGO stage and the CA125 level before initial treatment were significantly associated with the OS. In a multivariate analysis, the significance of the 2014 FIGO stage was preserved in the PFS of patients, whereas the significance of the CA125 level before initial treatment, LVSI and ITB disappeared. No independent variable was associated with OS.

**Table 2:** Univariate and multivariate survival analyses for progression-free survival (PFS) and overall survival (OS) of the patients with high-grade serous ovarian carcinoma.

Variables	Univariate PFS analysis	*P* value	Multivariate PFS analysis	*P* value
	HR (95% CI)		HR (95% CI)	
Age (< 60 years vs. ≥ 60 years)	1.111 (0.654–1.888)	0.697		
2014 FIGO stage (I and II vs. III and IV)	8.101 (2.511–26.140)	< 0.001	6.698 (1.890–23.732)	0.003
CA125 level before initial treatment*	1.936 (1.126–3.327)	0.017	0.977 (0.552–1.727)	0.935
Architectural grade (1 and 2 vs. 3)	0.717 (0.409–1.258)	0.246		
Nuclear grade (2 vs. 3)	1.302 (0.518–3.274)	0.575		
Intra-tumoral Budding (presence vs. absence)	1.452 (1.093–1.928)	0.010	1.094 (0.804–1.488)	0.568
Lymph vascular space invasion (presence vs. absence)	1.909 (1.085–3.359)	0.025	1.338 (0.736–2.434)	0.340
Mitoses (≤ 12 vs. > 12)**	0.741 (0.417–1.320)	0.309		
Lymph node metastasis (presence vs. absence)	1.368 (0.723–2.588)	0.336		
p53 expression (overexpression vs. complete absence)	0.902 (0.503–1.618)	0.729		

Variables	Univariate OS analysis	*P* value	Multivariate OS analysis	*P* value
	HR (95%CI)		HR (95%CI)	
Age (< 60 years vs. ≥ 60 years)	0.891 (0.449–1.767)	0.741		
2014 FIGO stage (I and II vs. III and IV)	8.392 (1.146–61.472)	0.036	4.927 (0.570–42.608)	0.147
CA125 level before initial treatment*	2.283 (1.082–4.819)	0.030	1.516 (0.700–3.282)	0.291
Architectural grade (1 and 2 vs. 3)	0.591 (0.280–1.245)	0.167		
Nuclear grade (2 vs. 3)	0.903 (0.317–2.571)	0.848		
Intra-tumoral budding (presence vs. absence)	1.463 (0.984–2.176)	0.060	1.173 (0.773–1.781)	0.453
Lymph vascular space invasion (presence vs. absence)	1.409 (0.691–2.873)	0.346		
Mitoses (≤ 12 vs. > 12)**	0.608 (0.293–1.264)	0.183		
Lymph node metastasis (presence vs. absence)	1.055 (0.431–2.584)	0.907		
p53 expression (overexpression vs. complete absence)	0.886 (0.390–2.010)	0.772		

*HR* hazard ratio, *CI* confidence interval.

*Median, 680.0 U/ml (≤ 680.0U/ml vs. > 680.0 U/ml).

**X 10 high power fields.

Among the 13 patients treated with NAC, the prognostic statuses were dead of disease in 12 patients and alive with disease in 1 with median follow-up of 46 (range 12 to 63) months.

Among the four patients with LGSOC, one patient was dead of disease at 75 months, one patient was alive with disease at 68 months, and two patients were alive with no evidence of disease at 47 and 52 months.

## Discussion

When we examined the presence of ITB as a maker of destructive stromal invasion of SOCs, it was found to be a significant poor prognostic indicator for the PFS of patients with HGSOC, but did not reach statistical significance for the OS of patients with HGSOC in the univariate analysis. In a multivariate analysis, ITB was not an independent prognostic indicator. The prognostic significance of ITB was considered to be strongly affected by the 2014 FIGO stage. The large size studies stratified by the 2014 FIGO stage may be recommended.

It is well-known that TBs are part of the tumor microenvironment and are associated with epithelial-mesenchymal transition (EMT)^8^. EMT is characterized by cytoskeletal rearrangements, cell motility and invasion, increased cell-associated proteolytic activity and reprogramming of the gene expression^15^. ITB was significantly associated with the incidences of LVSI and lymph node metastasis in the present study. Increasing evidence has highlighted a close relationship between EMT and chemoresistance in epithelial ovarian carcinoma (EOC)^16^. In one study, the SKOV-3 EOC cells were shown to trigger both EMT and chemoresistance after treatment by carboplatin^17^. ITB was found in all HGSOCs after NAC, except for in two complete regression cases, and was associated with a low number of mitoses of tumors and poor prognosis of patients.

The stromal-epithelial patterns of invasion in SBTs of the ovary have been subclassified as destructive and nondestructive. By definition, well-differentiated serous tumors featuring destructive stromal invasion are classified as LGSOCs whereas those with either no stromal invasion or stromal microinvasion are classified as SBTs of the ovary^18^. In a review, LGSOC was defined as a serous neoplasm showing destructive invasion, mild to moderate cytologic atypia, and relatively low proliferative activity. LGSOC accounts for 4.7% of SOCs and has an excellent prognosis, but long-term follow-up indicates that the prognosis for patients with stage III–IV disease is poor^19^. In the present study, LGSOC accounted for 4.8% of SOCs. ITB was found in one of four LGSOCs. The case number of LGSOC was too small to analyze the prognostic significance.

Analyses of gene expression microarray data from The Cancer Genome Atlas (TCGA) project revealed that HGSOC could be classified as one of four gene expression subtypes: mesenchymal, immunoreactive, proliferative, or differentiated^20^. Tumors with the mesenchymal phenotype had poor prognoses, whereas those with the immunoreactive type had favorable prognoses. Murakami et al.^21^ said that a feature of spindle and isolated cells with destructive stromal reaction was referred to as the mesenchymal phenotype. Tothill et al.^22^ reported that a novel subtype of HGSOCs reflected a mesenchymal cell type, characterized by the overexpression of N-cadherin and P-cadherin and low expression of differentiation markers, including CA125 and MUC1. A poor prognosis subtype was defined by a reactive stroma gene expression signature, correlating with extensive desmoplasia in such samples.

The presence of morphological HGSOC showing wild type of p53 expression is controversial. One study reported that wild type of p53expression was observed in nine (5.3%) HGSOCs of which two (1.2%) did not have a detectable TP53 mutation^7^. Chui et al.^23^ said that the existence of TP53-wildtype HGSCs is rare and comprise a heterogenous group of tumors which may arise via distinct pathogenetic mechanisms.

In conclusion, ITB was shown to be a cost-effective prognostic indicator for patients with HGSOC and a histopathologic biomarker of the progression of HGSOC. We may need to pay closer attention to the progression of HGSOCs.

## Materials and methods

### Case selection

After a review of the 318 malignant ovarian tumors found in the database of the Department of Pathology at the University of Occupational and Environmental Health Hospital between 2000 and 2017, 84 SOCs were selected. Twenty peritoneal carcinomas, defined according to the Gynecological Oncology Group inclusionary criteria^24^, were not included in the present study. The ovarian specimens were taken from 71 patients at primary surgery and 13 after neoadjuvant chemotherapy (NAC). All cases with available histopathologic slides were included in the present study, except for two patients with complete regression of SOC after NAC. Hematoxylin and Eosin (H&E)-stained slides of the SOCs were cut from a median of 6 (range 2–18) tissue blocks per case.

Ethical approval for this study was granted by the Review Board of the University Hospital of Occupational and Environmental Health on Ethical Issues (H27-185). All methods were carried out in accordance with relevant guidelines and regulations. Informed consent was obtained from all participants.

The following clinicopathological parameters were evaluated: age at the diagnosis, CA125 level (before initial treatment and after three cycles of chemotherapy), 2014 FIGO stage, architectural grade, nuclear grade, ITB, lymphovascular space invasion (LVSI), mitoses and lymph node metastasis while referring to previous studies^1,3,25^. The postsurgical assessment of the interval debulking surgery was available for the 2014 FIGO stage of patients who received platinum-based NAC. Adjuvant platinum-based chemotherapy was not performed in one patient with stage IA HGSOC without ITB, and six patients with severe medical complication and/or advanced age, (including two with HGSOC without ITB and four with HGSOC with ITB). These latter six patients were not included in the analysis of the patient prognosis.

According to the statements of the international tumor budding consensus conference (ITBCC)^11^, ITB was defined as a single tumor cell or a cell cluster of up to four tumor cells at the invasive tumor front. ITB was evaluated in the surgical specimens of the ovarian tumors to be clinically the site of the greatest tumor volume/size.

While we initially attempted to use a three-tier grading system of ITB (none, focal and diffuse) with a reference of Silva system for invasive endocervical adenocarcinomas^26^, there was no significant difference in the cumulative survival between women with SOC with focal and diffuse ITB. The SOCs were therefore ultimately divided into two groups: (those with and without ITB).

### p53 immunohistochemistry

Representative formalin-fixed paraffin-embedded (FFPE) tissue blocks of 84 cases were selected for immunohistochemical analysis. Immunohistochemistry for p53 was performed on formalin-fixed paraffin-embedded tissue sections using a commercially available mouse monoclonal anti-human antibody (Protein Clone DO-7, Dako, CA, USA) at a dilution of 1 in 50. The sections were stained by use of the universal immunoperoxidase polymer method (Envision kit; Dako), in accordance with the manufacturer’s instructions. They were subsequently subjected to microwave heating and pressure cooking for the purpose of antigen retrieval. Positive and negative controls were also established. According to the proposed immunohistochemical scoring^7^, expression of p53 was scored as overexpression, complete absence. cytoplasmic or wild type.

### Statistical analyses

Statistical analyses were carried out using the IBM SPSS Statistics, version 27 (IBM SPSS Statistics for Windows, IBM Corporation, Armonk, NY, USA). Data for the clinicopathological factors were evaluated using the Chi-square test or Mann–Whitney *U* test. The progression-free survival (PFS) was defined as the time from the date of initial treatment to the date of objective disease progression or last follow-up. The overall survival (OS) was defined as the time from the date of initial treatment to the date of death or last follow-up. The PFS and OS curves were estimated using the Kaplan–Meier method and compared using the log rank test. Univariate and multivariate Cox proportional hazards models were used to determine the association between potential risk factors and progression as well as death from disease. Statistical significance was considered to exist at a value of P < 0.05.

### Ethics approval

Ethical approval for this study was Granted by the Review Board of the University Hospital of Occupational and Environmental Health on Ethical Issues (H27-185).
